# The Role of Autophagy in Cancer Evolution and Prognosis, Highlighting Its Role in PCa and Its Interaction with Apoptosis and Epigenetic Regulation by miRNAs

**DOI:** 10.3390/medsci14040471

**Published:** 2026-08-10

**Authors:** Magdalena Kurkiewicz, Aleksandra Moździerz, Anna Rzepecka-Stojko, Jerzy Stojko

**Affiliations:** 1 Department of Toxicology, Toxicological Analysis and Bioanalysis, Faculty of Pharmaceutical Sciences in Sosnowiec, Medical University of Silesia in Katowice, 41-200 Sosnowiec, Poland; 2 Faculty of Medicine, Branch in Bielsko-Biała, Medical University of Silesia in Katowice, 43-382 Bielsko-Biała, Poland; 3 Department of Drug and Cosmetics Technology, Faculty of Pharmaceutical Sciences in Sosnowiec, Medical University of Silesia in Katowice, 41-200 Sosnowiec, Poland

**Keywords:** prostate cancer, autophagy, apoptosis, miRNA (microRNA), molecular markers, targeted therapy, treatment resistance, hydroxychloroquine

## Abstract

Background: Autophagy is a process that diversely impacts the stages of both tumor initiation and progression. Elucidating the molecular mechanisms underlying autophagy and its role in tumorigenesis is a key component of anticancer strategies in both prostate cancer and other malignancies. Because advanced prostate cancer frequently exploits enhanced autophagy as a defense mechanism against therapy-induced stress (e.g., from abiraterone), the pharmacological modulation of miRNA levels presents a tremendous opportunity to block the tumor’s escape route and overcome drug resistance. Methods: A comprehensive literature review was conducted to evaluate the molecular pathways determining cancer cell survival and death. The analysis focused on the dual nature of autophagy (functioning as a ‘double-edged sword’) within the tumor microenvironment, microRNA (miRNA) regulatory networks, and the efficacy of synergistic therapeutic strategies in overcoming treatment resistance. Results: The primary focus of this paper is the dual and complex role of autophagy, which serves, on the one hand, as a cellular protective shield against metabolic stress—thereby facilitating metastasis—and, on the other hand, as a potential pathway leading to autophagic cell death. The progression of this crucial process is regulated by intricate interactions (crosstalk) with apoptotic pathways, mediated by Bcl-2 family proteins, key kinases (such as mTOR, JNK, and DAPK), and transcription factors, such as p53. Furthermore, the autophagic machinery is precisely regulated by specific miRNA molecules (e.g., miR-21, miR-141, and miR-375). These not only act as crucial intracellular modulators of autophagy but also serve as promising circulating biomarkers, enabling the monitoring of this process’s activity throughout disease progression. Conclusions: Autophagy, and in particular its modulation via miRNA signaling networks, represents a major and highly promising translational target. By directly impairing this autophagic survival mechanism, ‘double-hit’ combination therapies—integrating autophagy inhibitors (such as hydroxychloroquine or VPS34 inhibitors) with standard antiandrogen or cytotoxic agents—demonstrate promising preclinical potential in overcoming treatment resistance and favorably modulating the immune microenvironment in advanced prostate cancer.

## 1. Introduction

The prostate gland is the organ most susceptible to carcinogenesis in males [1]. Survival rates among PCa patients depend on the tumor stage; however, early detection remains of paramount importance. The initial stages of the disease are potentially curable through surgery [2,3], whereas more advanced stages necessitate the administration of systemic treatments, such as androgen deprivation therapy (ADT), chemotherapy, and radiotherapy [2]. Despite the use of adjuvant chemo- or radiotherapy, PCa exhibits a high degree of treatment resistance [4,5]. Consequently, current research focuses on the strictly molecular mechanisms of carcinogenesis, targeting a multitude of cellular pathways, including apoptosis [6], ferroptosis [7], and autophagy [8].

The malignant transformation of the prostate is a multistep process that initiates with prostatic intraepithelial neoplasia, which can subsequently progress to metastatic prostate cancer [9]. The primary risk factors for prostate cancer development—aside from age, race, and environmental factors—include genetics, such as specific gene mutations (e.g., HOXB13, BRCA2, and CHEK2) and Lynch syndrome. Approximately 50% of prostate cancer cases involve gene rearrangements, with the TMPRSS2-ERG fusion being the most frequently reported. The TMPRSS2 gene is highly androgen-responsive, whereas the ERG gene is an oncogene belonging to the ETS family of transcription factors. In this context, the gene fusion leads to the overexpression of the ERG oncogene, thereby promoting cellular invasiveness and influencing further pro-tumorigenic processes, including extracellular matrix degradation and the epithelial-mesenchymal transition (EMT) [10]. The loss of tumor suppressors and alterations in the PI3K/AKT/mTOR signaling pathway also constitute major drivers of tumor progression. The loss of the PTEN gene, a master regulator of the PI3K/AKT/mTOR pathway, results in the hyperactivation of this cascade, thereby accelerating uncontrolled proliferation and inhibiting cancer cell death [11]. Defects in the TP53 and RB1 genes are equally significant; given their critical roles in cell cycle regulation, their concurrent inactivation is predominantly observed in a highly aggressive variant of the disease: neuroendocrine prostate cancer (NEPC) [12]. Alterations in DNA damage repair (DDR) pathways are also crucial contributors to prostate cancer development. Advanced prostate cancers (accounting for approximately 20–30% of these malignancies) harbor innate or acquired defects in genes responsible for DNA repair, most notably BRCA1, BRCA2, and ATM [13].

Carcinogenesis is further driven by modifications within tumor suppressor gene promoters and the dysregulation of miRNA molecules. In this context, miRNAs function either as oncogenes that facilitate cancer cell survival or as silenced tumor suppressors [14]. However, the androgen receptor (AR) plays the most pivotal role in prostate cancer. Depending on the availability of testosterone, its activation or inhibition directly impacts multiple signaling cascades, including mTOR, which serves as a fundamental regulator of autophagy. In addition to its interaction with the mTOR kinase, the androgen receptor directly crosstalks with ATG genes, which are crucial for the autophagic machinery. Furthermore, androgen receptor blockade alters intracellular ATP levels, indirectly affecting mTOR activity and thereby modulating autophagy [15].

Autophagy exerts a dual, context-dependent role in PCa, paradoxically functioning as both a suppressor of early tumorigenesis and a driver of advanced tumor cell proliferation, survival, and metastatic dissemination. Given the intricate molecular crosstalk between autophagic cascades and apoptotic signaling networks, the modulation of autophagic pathways significantly influences cancer cell plasticities and dictates the ultimate therapeutic efficacy of administered chemotherapy or radiotherapy [16]. Castration-resistant prostate cancer (CRPC) represents a more refractory malignancy that progresses independently of castrate levels of circulating testosterone (e.g., following androgen deprivation therapy) [17]. Because CRPC cells frequently develop alternative mechanisms for androgen receptor (AR) pathway activation and exhibit significant epigenetic alterations, it is imperative to elucidate the developmental mechanisms of this cancer at the level of DNA modifications. The pathogenesis of PCa involves, among other factors, DNA methylation and demethylation, histone modifications [18], and the RE1-Silencing Transcription factor (REST) [19].

This paper is intended as a narrative review. A comprehensive literature search was conducted to identify relevant studies investigating the role of autophagy, microRNAs and preclinical models in prostate cancer evolution and therapeutic resistance. The literature screening was performed across major international electronic databases, including PubMed, Scopus, Web of Science and Google Scholar. The final research was conducted on 8 July 2026, resulting in the inclusion of 142 relevant references, spanning primarily from 2010 to 2026, while also including foundational classic papers. Since this is a narrative review, a formal systematic-review process wa not applied, but strict inclusion and exclusion criteria were utilized to ensure relevance.

The search strategy employed combinations of the following Medical Subject Headings (MeSH) terms and keywords using Boolean operators (AND/OR): “prostate cancer”, “castration-resistant prostate cancer”, “CRPC”, “autophagy”, “microRNA”, “miRNA”, “apoptosis”, “enzalutamide”, “abiraterone resistance”, “autophagy inhibitors”, “chloroquine”, “ROC-325”, “preclinical models”, “patient-derived xenografts”, “PDX”, and “genetically engineered mouse models”.

To ensure the high quality and relevance of the gathered data, specific inclusion and exclusion criteria were applied during the literature selection process:

Inclusion criteria: (1) Peer-reviewed original research articles evaluating the molecular mechanisms of autophagy in prostate cancer; (2) Studies focusing on the epigenetic regulation of autophagic pathways by non-coding RNAs (miRNAs); (3) Preclinical in vitro and in vivo studies testing autophagy modulators or targeted drug delivery systems; (4) High-impact review articles and meta-analyses that provided critical thematic context. Only articles published in English and Polish were considered.

Exclusion criteria: (1) Conference abstracts, prospective trial registrations with no results, and editorials lacking original or pooled data; (2) Studies focusing on autophagy in non-prostatic malignancies without direct relevance to the discussed pathways; (3) Non-peer-reviewed preprints or gray literature.

A total of over 170 records were initially screened based on titles and abstracts, and the final selection of references was based on their methodological rigor and relevance to the translational focus of this review.

## 2. Molecular Pathways of Autophagy

Autophagy is a cellular degradation mechanism characterized by the formation of autophagosomes and the mediation of their fusion with lysosomes, facilitating the “recycling” of cellular components. Three primary types of this process are distinguished: chaperone-mediated autophagy (CMA), microautophagy, and macroautophagy [20].

Chaperone-mediated autophagy (CMA) is a selective process during which specific proteins containing a KFERQ-like motif are degraded [21]. These substrate proteins are recognized by the hsc70 chaperone and transported to the lysosomal surface, where they bind to the LAMP-2A receptor. This interaction induces the structural reorganization of LAMP-2A, facilitating the translocation of the unfolded protein into the lysosomal lumen for degradation by lysosomal enzymes [22]. This type of autophagy is most robustly activated under conditions of cellular stress (e.g., hypoxia, starvation) [23]. In cancer, this process exerts a dual role: insufficient CMA activity disrupts protein homeostasis and promotes carcinogenesis [24], whereas in an established tumor, its upregulation enhances cell survival and drives the Warburg effect [25]. Consequently, the targeted therapeutic modulation of these pathways in cancer cells may suppress tumor growth, restrict metastatic dissemination, and enhance chemosensitivity, a translational potential closely shared with emerging targeted strategies directed at alternative degradative routes such as microautophagic membrane dynamics [26].

Microautophagy involves the direct engulfment of cytoplasmic cargo via invaginations of the lysosomal membrane. This process is dependent on the cellular nutritional status and constitutes a multistep mechanism—relying on the formation of characteristic autophagic tubes (regulated by ESCRT complexes) and the subsequent pinching off of mature vesicles [27]. Once inside the lysosomal lumen, these vesicles are degraded by acid hydrolases, allowing the basic nutrients to be released back into the cytoplasm for recycling [28].

Macroautophagy is the most prominent and prevalent form of this process, characterized by the formation of transient, double-membrane vesicles (phagophores) that maintain intracellular homeostasis [29]. Nutrient deprivation or cellular stress inactivates the mTORC1 kinase, triggering the activation of the Atg13-ULK1-FIP200 complex and its localization to the omegasome [30]. Subsequently, mediated by the class III PI3K complex and Atg9A-containing vesicles, the membrane elongates and seals to form a mature autophagosome. Two essential ubiquitin-like conjugation systems govern this process: the Atg12-Atg5 complex and the Atg8/LC3 system [31]. Mature autophagosomes then traffic towards and fuse with lysosomes to form autolysosomes. Within these structures, lysosomal hydrolases degrade the engulfed cargo into basic amino acids, which the cell can subsequently reuse [32].

### 2.1. Autophagy and Prostate Cancer

The role of autophagy in PCa is frequently characterized as a “double-edged sword” [16]. On the one hand, it functions as a mechanism that promotes cancer cell metastasis; on the other hand, under specific and well-defined conditions, it can inhibit both carcinogenesis and metastatic spread. The ultimate impact of autophagy on the fate of prostate cancer cells is determined by complex protein interactions, which can be categorized into several key processes.

#### 2.1.1. AR- Dependent TFEB Expression and Enhancement of PCa Cell Proliferation

Enhancement of PCa cell proliferation: This process is associated, inter alia, with the transcription factor EB (TFEB), whose role in autophagy involves the regulation of lysosomal function. Autophagy is of significant importance in androgen-dependent PCa, as the androgen receptor (AR) is responsible for stimulating TFEB expression to induce the autophagic response. Furthermore, the AR regulates other mediators of autophagy—such as Atg4B, Atg4D, ULK1, and ULK2—thereby driving tumor cell progression [33].

#### 2.1.2. HIF-1α-Mediated Auophagy Induction in the Tumor Microenvironment

HIF-1α-mediated induction: Another process driving the proliferation of prostate cancer cells is the induction of autophagy via the activation of HIF-1α (hypoxia-inducible factor 1-alpha), which subsequently binds to the promoter of the ATG5 gene (a core autophagy regulator). In PCa, under normoxic conditions, the HIF-1α factor plays a critical role in regulating both chemoresistance and radioresistance. Under hypoxic conditions within the tumor microenvironment, HIF-1α induces the transcription of the BNIP3 gene. The BNIP3 protein subsequently binds to the apoptotic pathway proteins Bcl-2 and Bcl-xL, which interact with Beclin-1, an essential autophagy regulator. The competitive binding of BNIP3 to Bcl-2 and/or Bcl-xL attenuates their interaction with Beclin-1, leading to its dissociation and the subsequent activation of autophagy [34].

#### 2.1.3. Pro-Autophagic Role of FGF21 Signaling

FGF21 signaling: Another pro-autophagic factor involves fibroblast growth factor 21 (FGF21), which participates in the inhibition of the PI3K/Akt/mTOR signaling pathway (a critical cascade in prostate cancer). Overexpression of FGF21 enhances the conversion of LC3-I to LC3-II, while the expression of LAMP2 decreases p62 levels and increases autophagosome formation in LNCaP cells, suggesting that FGF21 may facilitate autophagic flux [35]. However, it should be noted that steady-state changes in LC3-II or p62 levels alone do not definitively demonstrate increased autophagic flux without further validation using lysosomal inhibition assays or tandem fluorescent reporters.

#### 2.1.4. Regulation of Autophagy and CMA by the PI3K/Akt/mTOR Axis

The mTOR kinase, a primary mediator of the PI3K/Akt pathway, is intrinsically linked to autophagy regulation. The suppression of autophagy resulting from the aberrant activation of mTORC1 may contribute to carcinogenesis. In experiments utilizing mice with a targeted deletion of the PTEN (phosphatase and tensin homolog) gene, a reduced incidence of autophagy was observed. While autophagy exerts a tumor-suppressive effect on cancer cells during the early stages of carcinogenesis, it is paradoxically upregulated in advanced-stage tumors to satisfy their elevated energetic requirements [36,37].

Recent studies have demonstrated the regulation of chaperone-mediated autophagy (CMA) directly on the lysosomal surface via the mTOR/Akt/PHLPP pathway. The phosphorylation of Akt by the mTORC2 complex sustains the inhibitory effect of Akt kinase on CMA. Maximal activation of this specific type of autophagy is achieved through the active, Rac1-dependent recruitment of PHLPP phosphatase to the lysosomal membrane, followed by the dephosphorylation of Akt. Current experiments have also revealed that the PI3K/Akt/mTOR signaling pathway exerts an anti-apoptotic effect and fundamentally contributes to the regulation of autophagy in tumor cells [38].

#### 2.1.5. MAPK8/JNK1 Inhibition and Cytotoxic Autophagy

Reduction of proliferation and cytotoxic autophagy: The MAPK8/JNK1 kinase frequently promotes the proliferation and survival of prostate cancer cells, rendering it an attractive target for oncological therapies. Although the JNK pathway is conventionally involved in the release of Beclin-1, it has been demonstrated that the pharmacological inhibition of MAPK8/JNK1 can induce a phenomenon known as cytotoxic autophagy (ultimately leading to cell death) in tumor cells. The implementation of such targeted treatment drastically diminishes the proliferative capacity of the malignancy [39,40].

#### 2.1.6. Autophagy as a Pro-Death Mechanism via STK11/AMPK Signaling

Autophagy as a pro-death mechanism: Experimental models investigating the tumor protein D52 (TPD52/PrLZ/prostate leucine zipper), encoded on chromosome 8q21.1, demonstrate that inhibiting its interaction with STK11/LKB1 (serine/threonine kinase 11) using docetaxel promotes the unhindered expression and activity of STK11. As an upstream mediator of the AMPK signaling pathway, STK11 consequently induces autophagy, which subsequently triggers apoptosis in cancer cells. Furthermore, it has been established that STK11/LKB1 expression is progressively lost during the transition from normal prostatic tissue to malignancy. Functionally, AMPK activity exhibits extensive crosstalk with the phosphoinositide 3-kinase (PI3K), mTOR, and mitogen-activated protein kinase (MAPK) signaling pathways [41].

## 3. Autophagy and Apoptosis

Autophagy and apoptosis represent two distinct mechanisms of cellular response to adverse environmental conditions. The induction of autophagy serves as an adaptive strategy to prevailing stressors, facilitating cell survival and the evasion of death. In contrast, apoptosis enables the complete neutralization of cells that pose a threat to the integrity of normal tissues [42].

Prostate cancer cells exhibit an extensive functional crosstalk between autophagy and apoptosis. A pivotal factor in this context is the tumor necrosis factor-related apoptosis-inducing ligand (TRAIL), which triggers apoptosis by binding to specific death receptors (DR4 and DR5) and recruiting the adaptor protein FADD alongside caspase-8, thereby assembling the death-inducing signaling complex (DISC) [43]. TRAIL operates synergistically: it robustly stimulates apoptotic pathways while simultaneously inducing autophagy in prostate cancer cells, which acts as a cytoprotective mechanism during the initial stages of exposure to adverse conditions [44].

Autophagy can also promote apoptosis by actively directing the cell toward death. Core autophagy-related proteins exert a stimulatory effect on the activation of apoptotic pathways; notably, Atg5 and Atg12 are implicated in this process, and their depletion effectively silences caspase activity. Furthermore, autophagy can promote apoptosis through the degradation of anti-apoptotic factors or cell survival proteins, such as inhibitors of apoptosis proteins (IAPs). The activation of autophagy-related pathways can indirectly impact mitochondrial integrity. For instance, the cleaved form of the Atg5 protein can translocate to the mitochondria, where it interacts with Bcl-2 family proteins. This interaction promotes the opening of the mitochondrial permeability transition pore and precipitates the apoptotic signaling cascade [45,46]. In an experimental model utilizing mouse embryonic fibroblasts (MEFs) treated with a sphingosine kinase inhibitor (SKI), the compound promoted cell death by inducing the translocation of the caspase-8 complex, Fas receptor, and the FADD adaptor protein to Atg5- and Atg16-positive autophagosomal membranes, thus facilitating the assembly of the apoptotic DISC complex [47].

Conversely, the inhibition of apoptosis by autophagy is primarily mediated through the suppression of activation or the complete degradation of pro-apoptotic proteins and executioner caspases. For example, the selective autophagic degradation of caspase-8 confers apoptosis resistance in certain malignancies, such as colorectal cancer [48].

### 3.1. The Role of Kinases in Modulating Both Mechanisms

The molecular effectors governing both apoptosis and autophagy encompass, among others, the Bcl-2 protein family, mitogen-activated protein kinases (MAPKs, e.g., JNK, p38), and transcription factors of the p53 family. The Bcl-2 family constitutes the fundamental regulatory core for programmed cell death. Crucially, the structure of certain Bcl-2 members exhibits homology with the autophagy initiator Beclin-1. Beclin-1 contains three distinct structural domains, including a BH3 domain. The presence of this domain classifies Beclin-1 as a “BH3-only” protein, enabling its direct binding to the hydrophobic binding groove of anti-apoptotic Bcl-2 proteins, which consequentially inhibits autophagy. The other two domains are the coiled-coil domain (CCD) and the evolutionarily conserved domain (ECD). The ECD is responsible for autophagy regulation and the suppression of tumorigenesis. Additionally, a short, leucine-rich sequence functions as a nuclear export signal (NES), ensuring proper subcellular localization [49,50]. Furthermore, Bcl-2 regulates autophagy by interacting with intermediate regulatory proteins, such as AMBRA1. Upon the reception of autophagic stimuli, AMBRA1 undergoes phosphorylation and translocates to the mitochondrial membrane. The interaction between Bcl-2 and AMBRA1 exerts an inhibitory effect on autophagy initiation by preventing the assembly of the core initiation complex. Studies conducted on the DU145 cell line (an androgen-independent prostate cancer model) have demonstrated that elevated AMBRA1 expression positively correlates with tumor advancement, as quantified by the Gleason score, highlighting the potential of autophagy-related proteins as prognostic indicators for prostate cancer progression [51,52].

Recent studies indicate that Beclin-1 lacks intrinsic pro-apoptotic activity; however, it is susceptible to the action of Bcl-2 or Bcl-xL proteins, which exert an inhibitory effect on autophagy upon binding to it. To initiate autophagy, the interaction between Beclin-1 and the anti-apoptotic Bcl-2/Bcl-xL proteins must be disrupted. This dissociation can occur via several mechanisms: the competitive displacement of Beclin-1 by other BH3 domain-containing proteins, the phosphorylation of the Bcl-2 protein mediated by JNK1 or ERK kinases, or the dysfunction of the NAF-1 (nutrient-deprivation autophagy factor-1) protein, which under physiological conditions stabilizes this inhibitory complex. The release of Beclin-1 may also result from its self-interaction and the formation of large oligomers, which become stronger, competitive targets for Bcl-2 and Bcl-xL proteins, thereby “displacing” free Beclin-1 and rendering it available for autophagy induction [53,54].

Within this regulatory axis, Bcl-2 family proteins govern autophagic and apoptotic responses in a parallel manner, typically suppressing both pathways. Notable exceptions include the pro-apoptotic BAX and BIM proteins, which accelerate cell death while actively repressing autophagy, and Beclin-1, which functions strictly as a pro-autophagic factor lacking intrinsic apoptotic capabilities [55]. Concurrently, the mTOR kinase, a master serine/threonine kinase, acts as a primary negative regulator of autophagy; under nutrient-rich or pro-survival conditions, hyperactivation of the mTORC1 pathway suppresses autophagic initiation. In the context of cell death, mTOR-mediated phosphorylation of BAD prevents its binding to anti-apoptotic Bcl-2 members, thereby simultaneously inhibiting apoptosis [56].

**Figure 1:**
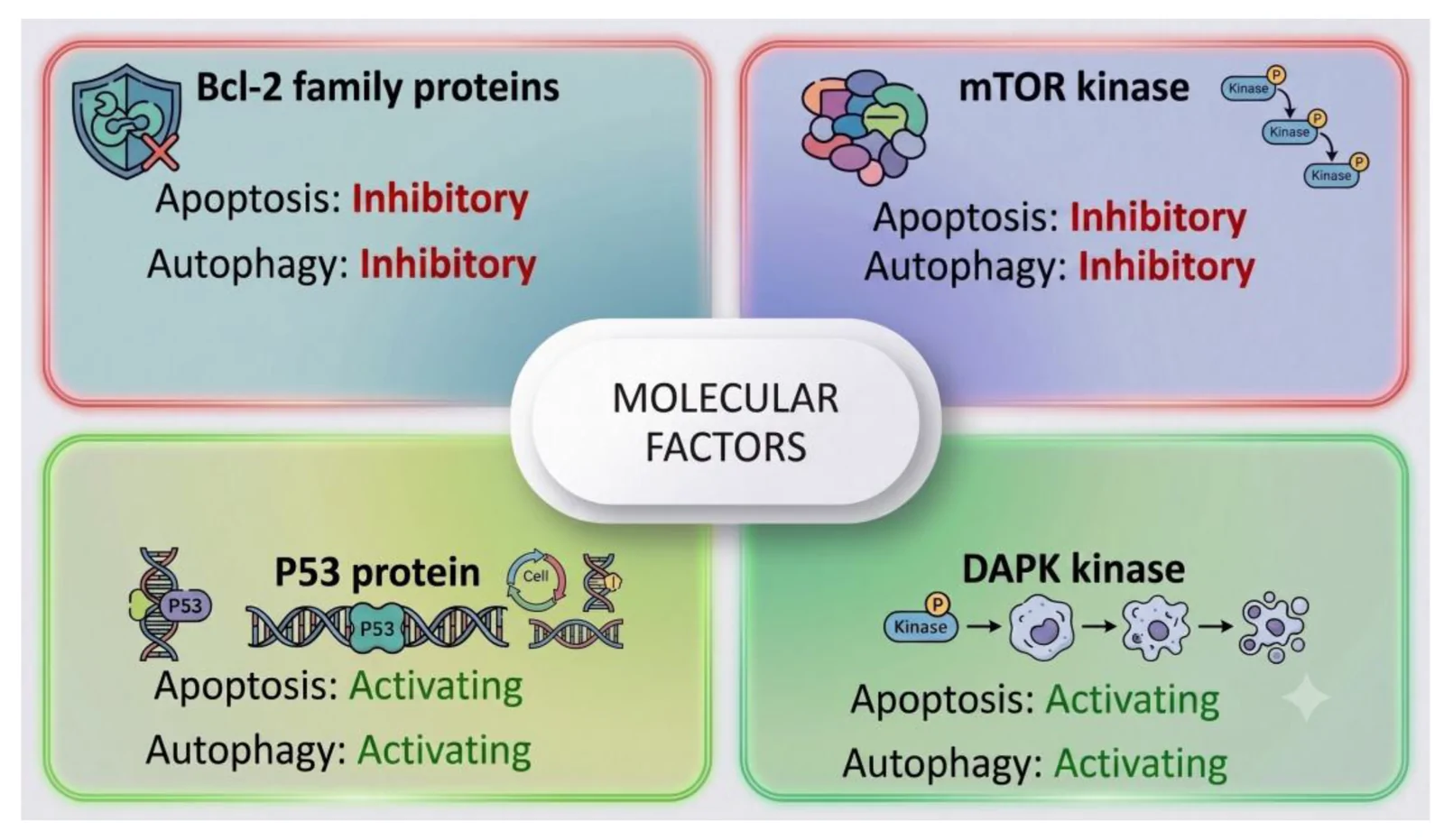
A schematic comparison of the impact of key molecular factors on the processes of apoptosis and autophagy. The upper section (panels with red borders) illustrates inhibitory factors, while the lower section (panels with green borders) presents activating factors. The attached icons graphically represent the nature or mechanism of action of individual molecules. Numbers in square brackets refer to the source literature. Graphics made using Google Gemini (Gemini 1.5 Pro) [59,60,61,62].

Furthermore, mitogen-activated protein kinases (MAPKs), particularly JNK, play a pivotal role under cellular stress. JNK-mediated phosphorylation of Bcl-2 disrupts its inhibitory interaction with Beclin-1 to trigger autophagy; however, during prolonged stress, sustained JNK activity drives the complete dissociation of Bax from Bcl-2, shifting the balance toward apoptotic execution [53,55]. This response is tightly coordinated with the tumor suppressor p53, which transcriptionally upregulates core autophagic genes (e.g., Atg4A, Atg5, ULK1/2) and activates downstream AMPK signaling while concurrently repressing anti-apoptotic targets [57]. Finally, death-associated protein kinase (DAPK) serves as a critical molecular switch; under starvation, DAPK1 interacts with MAP1B microtubules to facilitate autophagosome maturation, while also phosphorylating Beclin-1 to actively prevent its entrapment by Bcl-xL, thereby enabling unhindered autophagic flux [58]. The complex regulatory networks, cross-talk, and opposing or collaborative influences of these core kinases, transcription factors, and Bcl-2 family proteins on both autophagic and apoptotic pathways are visually mapped out in Figure 1.

### 3.2. Autophagy Markers as Prognostic Indicators in Prostate Cancer

#### 3.2.1. Prognostic Significance of Beclin-1 and LC3B Expression

An analysis of prostate tissue biopsies encompassing varying degrees of carcinogenesis (benign prostatic hyperplasia [BPH], adjacent prostate tissue, and confirmed prostate carcinoma) demonstrated that the expression of Beclin-1 and LC3B proteins is significantly elevated in prostate cancer tissues compared to benign prostatic hyperplasia. Elevated levels of LC3B and Beclin-1 positively correlated with higher Gleason scores and poorer clinical prognoses [63]. While elevated levels of these markers correlate with poor prognosis, definitive conclusions regarding dynamic autophagic flux require complementary assays, such as utilizing bafilomycin A1 for lysosomal inhibition.

#### 3.2.2. ULK1 as an Indpendent Predictor of Biochemical Recurrence

ULK1 serves as an independent predictive factor for biochemical recurrence. Early-stage prostate cancer cells exploit high levels of ULK1 to rapidly initiate autophagy, thereby evading apoptosis induced by an adverse tumor microenvironment. As demonstrated by Miyake et al. [64], a robust ULK1 signal in patients following radical prostatectomy indicates a persistent, high potential for tumor progression, often correlating with a rapid elevation in prostate-specific antigen (PSA) levels during post-operative monitoring.

#### 3.2.3. Oncogenic Functions of p62 Accumulation in Tumor Progression

**Figure 2:**
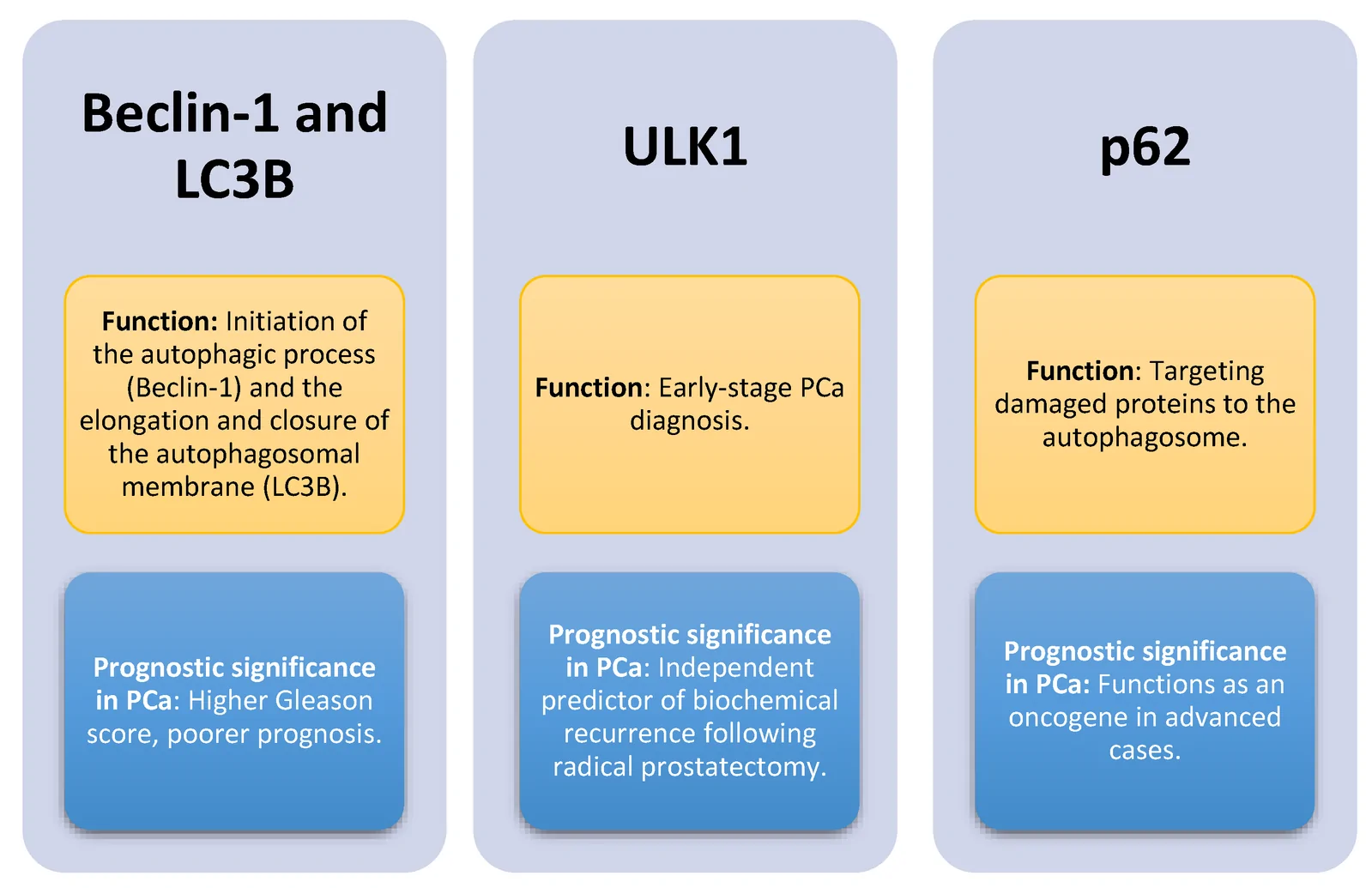
Selected autophagy biomarkers and their prognostic significance in prostate cancer based on data from [63,64,65].

Under physiological conditions, the primary function of the p62 protein is to direct damaged proteins and organelles to the autophagosome for degradation. However, in advanced prostate cancer, p62 accumulates to pathologically high levels and acquires oncogenic functions, promoting the epithelial-mesenchymal transition (EMT) and driving rapid tumor progression [31]. A summary of the prognostic significance of p62, alongside other key autophagy biomarkers such as Beclin-1, LC3B, and ULK1, is presented in Figure 2 [63,64,65].

## 4. The Role of Non-Coding RNA

The regulatory profiles, specific oncogenic or tumor-suppressive functions, and primary downstream molecular targets of key microRNAs (miRNAs) have been strictly validated in prostate cancer pathogenesis.

**Figure 3:**
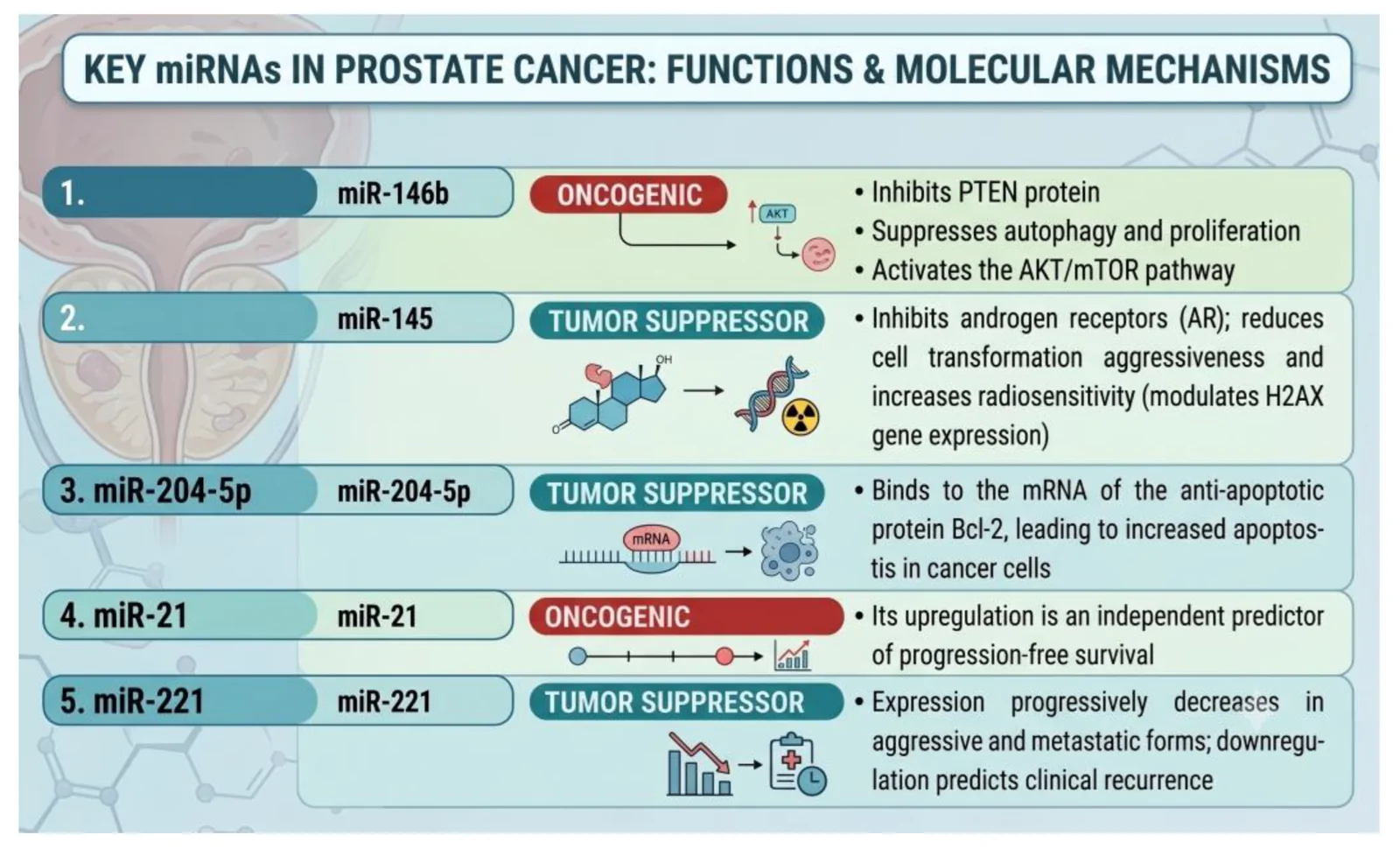
Key miRNAs in prostate cancer: their functional roles (oncogenic vs. tumor suppressive) and primary molecular mechanisms. The schematic was generated with the assistance of the Gemini AI model (Google) based on data from [71,72,73,74,75].

**Figure 4:**
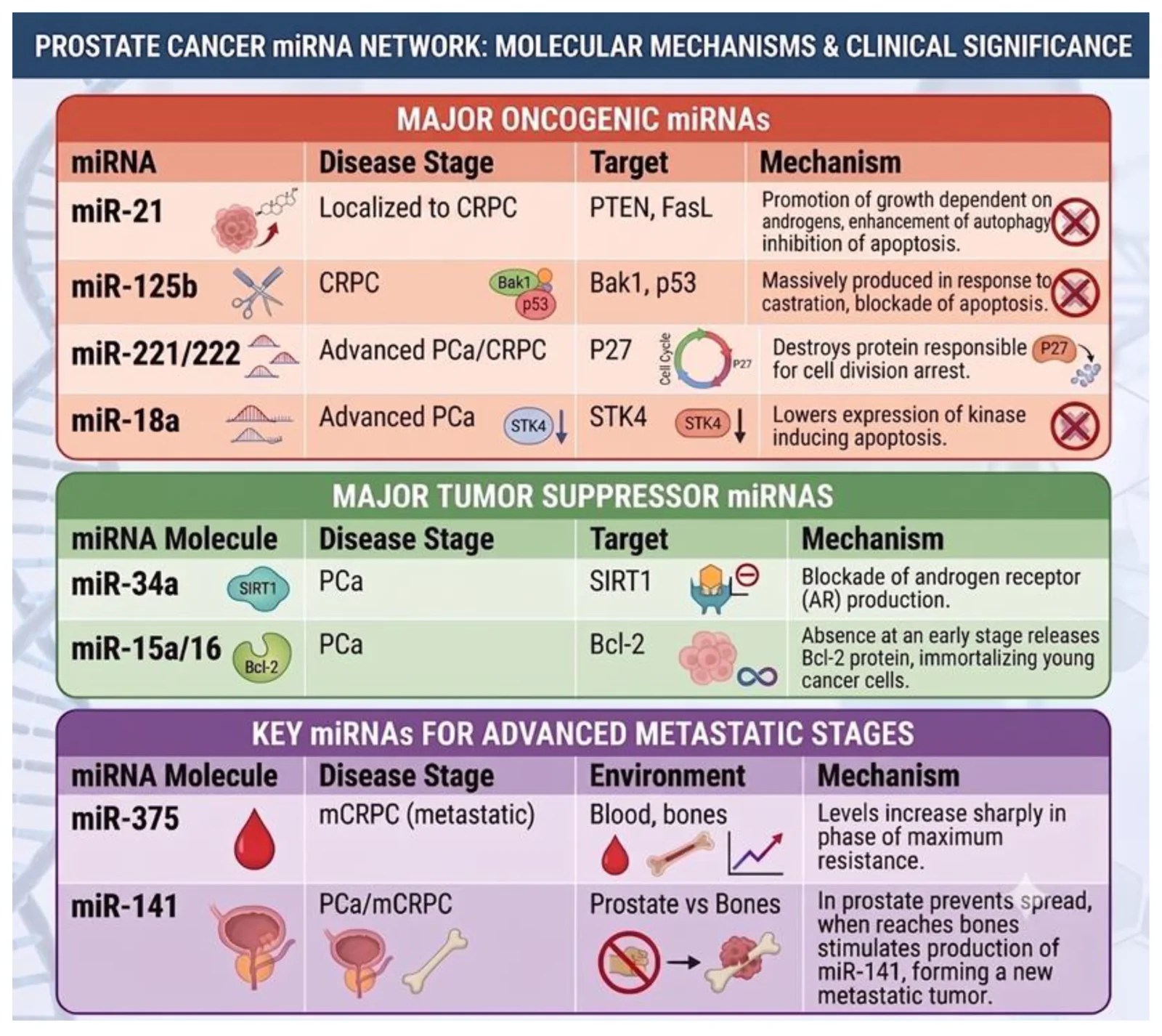
The prostate cancer miRNA network: molecular mechanisms and clinical significance across different disease stages. The schematic categorizes major oncogenic, tumor-suppressive, and metastasis-associated miRNAs (including mCRPC), highlighting their specific targets and cellular mechanisms. Generated with the assistance of the Gemini AI model (Google) based on data from [74,75,76,77,78,79,80,81,82].

MicroRNAs (miRNAs) are single-stranded RNA molecules of approximately 22 nucleotides in length, generated through a multi-step biogenesis pathway that encompasses several core stages: transcription of their respective genes to generate primary miRNA (pri-miRNA) precursors, maturation driven by Drosha and Dicer enzymes, nuclear export, and the final incorporation of the mature guide strand into the RNA-induced silencing complex (RISC) [66,67,68]. miRNAs are implicated in a multitude of cellular processes, predominantly in the regulation of proliferation, cellular differentiation, apoptosis, and embryogenesis. In the context of carcinogenesis, they play a highly significant role, possessing the capacity to act as either tumor suppressors or oncogenes (oncomiRs) [69,70]. The regulatory profiles, specific oncogenic or tumor-suppressive functions, and primary downstream molecular targets of key miRNAs strictly validated in prostate cancer pathogenesis are comprehensively depicted in Figure 3 [71,72,73,74,75]. To facilitate clinical translation and guide future therapeutic development, a structured framework categorizing major oncogenic, tumor-suppressive, and advanced metastatic miRNAs according to specific clinical disease stages is systematically organized in Figure 4 [74,75,76,77,78,79,80,81,82].

It is increasingly established that miRNAs function as negative regulators of gene expression by annealing to complementary sequences on target mRNAs, resulting in translational repression and mRNA degradation. In scenarios of miRNA overexpression (which may occur, for instance, due to the amplification of the genomic locus encoding a specific miRNA) targeting a tumor suppressor mRNA, the formation of this repressor complex leads to the downregulation of the tumor suppressor, thereby exacerbating cellular proliferation and driving malignant transformation processes. A parallel oncogenic effect can be induced by the downregulation of a specific miRNA responsible for silencing oncogenes, which consequently leads to elevated oncogene expression levels. This attenuation of miRNA expression may be attributed to chromosomal deletion or the epigenetic methylation of the locus encoding the given miRNA [83,84].

miRNAs participate in the regulation of both intrinsic and extrinsic apoptotic pathways, a factor of critical importance in oncological research and in strategies aimed at directing cancer cells toward programmed death. Depending on the specific molecule, miRNA action within the intrinsic pathway centers on modulating proteins such as Bax, Bak, Bcl-xL, and Bcl-2. Within the extrinsic pathway, established molecular targets for miRNAs include the FasL domain, the TRAIL receptor, DR4, c-FLIP, and FADD [85]. Key apoptotic regulators that are also subject to miRNA-mediated control predominantly include DAPK (a pro-apoptotic factor and metastasis suppressor), p53, and XIAP (an X-linked inhibitor of apoptosis protein) [86].

Discrepancies in recent studies suggest that the role of miRNAs remains somewhat ambiguous; miRNA-mediated regulation in carcinogenesis requires further investigation and a paradigm shift toward more definitive conclusions. To date, it has been demonstrated that:

- Guan et al. [87] demonstrated that the upregulation of miR-21 serves as an independent predictor of progression-free survival in patients diagnosed with advanced prostate cancer (PCa).
- The downregulation of miR-221 has been observed in patients experiencing prostate cancer recurrence. Spahn et al. [88] demonstrated that miR-221 expression progressively decreases in aggressive and metastatic prostate cancer, rendering it a predictive biomarker for clinical relapse.
- The ectopic introduction of miR-145 into prostate cancer cells (22Rv1) exerts an inhibitory effect on androgen receptors and attenuates the transformation of tumor cells into a more aggressive phenotype. Furthermore, miR-145 expression is significantly elevated in patients who maintain an annual PSA level of <2.0 ng/mL, despite the confirmed efficacy of implemented neoadjuvant radiotherapy (RT). Conversely, the expression of the H2AX gene, which is negatively regulated by miR-145, was found to be significantly reduced. Gong et al. [89] concluded that miR-145 modulates tumor radiosensitivity in prostate cancer.
- Elevated expression levels of specific miRNAs, including miR-135b, miR-194, miR-222, and miR-125b, serve as predictive indicators for the risk of early prostate cancer recurrence, as evidenced by human prostate cancer miRNA profiling conducted by Tong et al. [90].
- The expression levels of miRNAs such as miR-205, miR-224, miR-21, miR-126, miR-34b, and miR-15/16 undergo progressive dysregulation during the course of metastasis to bones or lymph nodes [91].

To date, nearly 3000 miRNAs have been identified, hundreds of which are directly implicated in the regulation of autophagy. The induction of autophagic processes is governed by a diverse array of these non-coding RNAs. As a central master regulator of autophagy, the mTOR kinase has been recognized as a direct target for several miRNAs during the progression of autophagy, prominently including miR-199a-3p, miR-100, miR-128, miR-338-3p, miR-7, and miR-96 [92]. Contemporary miRNA research suggests that these molecules may serve as highly viable novel biomarkers or actionable therapeutic targets in various malignancies, including prostate cancer. A notable molecule extensively investigated in the context of PCa is miR-146b, the functional impact of which was examined by Gao et al. [93]. The experimental results indicate that the administration of an miR-146b mimic significantly diminishes PTEN protein levels while concurrently elevating the levels of phosphorylated AKT (p-AKT) and phosphorylated mTOR (p-mTOR). Furthermore, miR-146b was found to be upregulated in prostate cancer (PCa) tissues. The overexpression of miR-146b promotes the proliferation of PC3 cells, whereas its knockdown exerts an anti-proliferative effect. These oncogenic functions of miR-146b are driven by the suppression of PTEN, which subsequently leads to the inhibition of autophagy via the hyperactivation of the AKT/mTOR signaling pathway in prostate cancer cells [93].

The critical regulatory role within this molecular network is further corroborated by a study by Cai et al., which investigated the correlation involving breast cancer anti-estrogen resistance 4 (BCAR4). This research demonstrated that the long non-coding RNA (lncRNA) BCAR4 contributes to the direct transcriptional activation of Beclin-1. This transactivation subsequently increases the LC3-II to LC3-I ratio, thereby enhancing autophagic flux in prostate cancer cells [94]. In the context of prostate cancer, miRNAs such as miR-106b, miR-17, and miR-20b may be of significant clinical importance, alongside the upregulation of miR-30. Researchers have discovered that miRNAs and target mRNAs play a substantial role in mediating neuroendocrine (NE) transdifferentiation in LNCaP cells. Current evidence confirms that the miR-17 family—encompassing miR-106b, miR-106a, miR-20b, miR-17, and miR-20a—significantly contributes to NE transdifferentiation in PCa, acting in concert with miR-375 [95].

In the pathogenesis of neuroendocrine prostate cancer (NEPC), the miR-421/ATM signaling pathway plays a pivotal role in determining therapeutic resistance. Key molecular alterations driving the progression of this highly aggressive PCa variant involve the upregulation of miR-301a and miR-375, coupled with the downregulation of miR-106a. During the advanced, metastasis-promoting stages, miR-7, miR-10a, miR-143, and miR-145a are of profound importance. Studies utilizing the LNCaP prostate cancer cell line consistently demonstrate that miRNAs and their target mRNAs profoundly influence the transdifferentiation of cells toward the NEPC phenotype, underscoring the fact that the miRNA-mRNA interaction network critically dictates PCa plasticity and progression [96].

In the context of autophagy, the regulatory role of miRNAs extends to the ULK complex, a critical component of the initial autophagic cascades; class III PI3K (including Beclin-1); the LC3B isoform of the LC3 protein; Atg4D, Atg7, and Atg12; and VMP1, a transmembrane protein involved in phagophore formation. When analyzing the impact of miRNAs on autophagy, it is essential to consider the signaling pathways that these molecules can modulate under conditions of starvation, cellular stress, or hypoxia. The AMPK/mTORC1 axis serves as a crucial checkpoint for autophagy control, as it directly influences ULK complex assembly and autophagy initiation, a cascade tightly managed by generalized microRNA regulatory networks [97]. For instance, while target validation screening confirms that components like miR-148b interact directly with AMPKα subunits to alter chemosensitivity in related solid tumor models [98], parallel non-coding networks such as miR-99a and miR-119a-3p consistently target the downstream mTOR/c-Met pathways to restrict aggressive cellular invasive potential.

miRNAs also exert regulatory control over both the intrinsic and extrinsic apoptotic pathways within the cancer landscape. In the extrinsic pathway, miRNA action is closely linked to the Fas/FasL complex. Experimental modulations demonstrate that the overexpression of miR-467a can predispose cancer cells to rapid tumor growth by repressing the expression of Fas and Bax, whereas apoptotic progression can be exacerbated following the application of specific miRNA inhibitors [99]. Furthermore, while certain non-coding elements like miR-155 function as fundamental apoptotic switches predominantly evaluated in hematological malignancies [100], local prostatic networks are driven by highly specific target interactions. Within the intrinsic apoptotic pathway, one of the primary regulators is the Bcl-2 protein family, which plays a pivotal role in prostate cancer cell survival. Specifically, the 3′ UTR of the Bcl-2 mRNA serves as a direct target for miR-204-5p. The binding of this miRNA effectively downregulates Bcl-2 expression, thereby augmenting the downstream apoptotic cascade and executing cell death directly in PCa cell lines such as PC3 and LNCaP [101].

**Figure 5:**
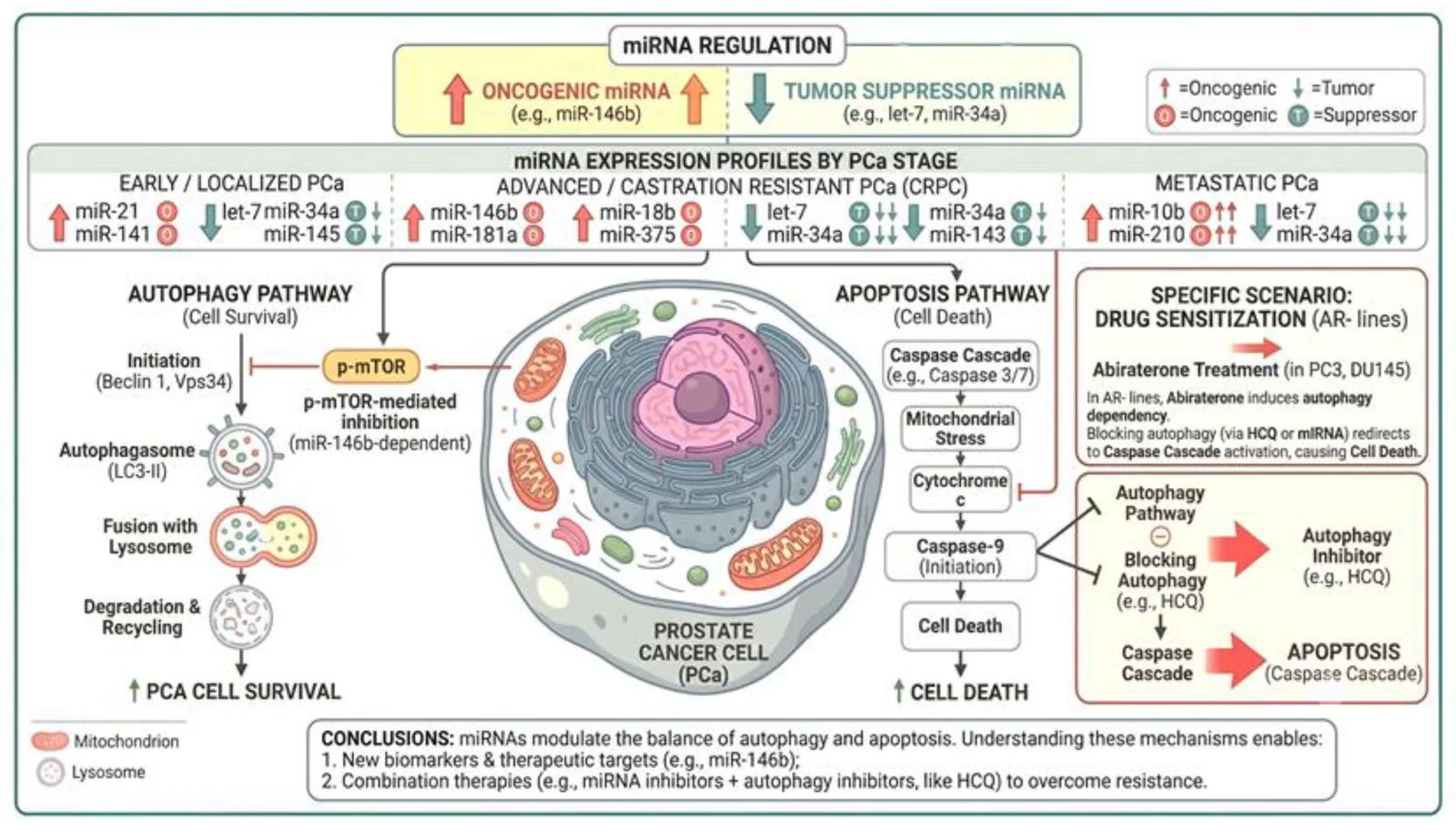
Comprehensive overview of miRNA-mediated regulation of autophagy and apoptosis in prostate cancer. The schematic outlines stage-specific miRNA expression profiles (localized, CRPC, and metastatic) and illustrates the molecular crosstalk between the cell survival (autophagy) and cell death (apoptosis) pathways. Furthermore, a specific therapeutic scenario demonstrates how drug-induced autophagy dependency during abiraterone treatment can be exploited; blocking autophagy (e.g., via HCQ) redirects AR-negative PCa cells toward the caspase cascade, ultimately leading to apoptosis. The schematic was generated with the assistance of the Gemini AI model (Google) based on data from [66,67,68,69,70,71,72,73,74,75,76,77,78,79,80,81,82,83,84,85,86,87,88,89,90,91,92,93,94,95,96,97,98,99,100,101,102]. It should be noted that the depicted molecular interactions include both direct physical bindings and indirect regulatory associations derived from preclinical models.

The most prevalent circulating miRNAs detected in the blood of patients diagnosed with prostate cancer include miR-21, miR-141, and miR-375. miRNAs play a paramount role in PCa pathogenesis due to their influence on the epithelial-mesenchymal transition (EMT), a critical step for the development of metastatic capabilities in prostate cancer cells. Specific miRNAs participate in regulating the transition of epithelial cells to a mesenchymal phenotype, acting either as suppressors (e.g., miR-200) or oncogenes (e.g., miR-34b) [102]. A comprehensive mechanistic overview of this miRNA-mediated epigenetic regulation, alongside a distinct translational scenario demonstrating cellular drug sensitization and the redirection of autophagy-dependent cells toward apoptosis during clinical treatment, is illustrated in Figure 5.

## 5. Clinical Correlations Between Prostate Cancer and Prior Autophagic/Apoptotic Pathways

Androgen deprivation therapy (ADT) is a form of hormonal therapy designed to reduce serum testosterone levels. A primary inhibitor of androgen biosynthesis, including testosterone, is abiraterone, which functions by blocking androgen synthesis via 17α-hydroxylase/lyase inhibition and exerting antagonistic effects on the androgen receptor (AR). Currently, our molecular understanding of prostate cancer resistance revolves around genomic alterations, intracellular signaling pathway modifications, and autophagy. Autophagy appears to act as a cytoprotective mechanism for prostate cancer cells; thus, targeting this process emerges as a potentially effective strategy for designing novel therapeutics based on AR antagonism. Mortezavi et al. demonstrated in standard PCa cell lines (LNCaP, DU145, and PC3) that treating the androgen-dependent LNCaP line with abiraterone in combination with autophagy inhibitors resulted in a profound decrease in cell viability, as evidenced by Annexin V assays confirming apoptosis. Autophagy inhibition was also noted in androgen-independent lines (DU145 and PC3)—following abiraterone treatment, their viability decreased, accompanied by the activation of the caspase cascade leading to cell death [103].

While the roles of microautophagy and chaperone-mediated autophagy (CMA) in prostate cancer remain poorly understood and require further investigation, macroautophagy is a highly documented mechanism in preclinical models of this malignancy. Current research indicates that autophagy plays a significant role in prostate cancer development. Utilizing an orthotopic mouse model with an enzalutamide-resistant C4-2B cell line, it was demonstrated that in response to androgen deprivation, prostate cancer cells are capable of initiating AMPK-dependent autophagy. In vivo, a combination therapy comprising enzalutamide and autophagy modulators significantly inhibited tumor growth compared to monotherapy, thereby proving the efficacy of autophagy blockade [104]. Orthotopic xenograft models of metastatic castration-resistant prostate cancer (mCRPC) are also developed to investigate other mechanisms. A study by Zhao et al., which analyzed autophagy as a response to the tumor microenvironment, indicates that vascular endothelial cells secrete chemokines responsible for suppressing androgen receptor (AR) expression, thereby forcing the cells to undergo autophagy. Consequently, autophagy enabled the cells to migrate and metastasize, and its inhibition significantly reduced their metastatic capacity [105].

Synergy with targeted therapies and patient-derived xenograft (PDX) models in experimental studies further substantiate the effectiveness of autophagy inhibition. In research conducted by Butler et al., in addition to in vitro cell lines, tumor tissue fragments obtained directly from a patient were implanted into mice to preserve natural heterogeneity and the appropriate tumor microenvironment. It was shown that upon AKT/mTOR inhibition, the PDX model tissue initiates not only autophagy but also alternative survival pathways (Ras/MEK/ERK), which explains the failure of monotherapy [106]. Furthermore, during the investigation of a promising novel plant-derived anticancer agent in immunodeficient CD-1 mice engrafted with PC3 prostate cancer cells, it was demonstrated that the administration of this compound induces autophagy. The addition of chloroquine as an autophagy inhibitor sensitized the tumors to the therapy by suppressing the Ki67 proliferation marker and stimulating apoptosis [107].

The protective role of autophagy and metabolic stress cooperation can be further exploited clinically; for instance, modern strategic combinations evaluating antiandrogens with metabolic modulators aim to systematically overcome systemic autophagy-mediated therapeutic resistance in castration-resistant cohorts during advanced clinical trial settings [108].

Monitoring autophagic pathways is equally critical during the administration of radiotherapy. The primary objective of radiotherapy is to induce DNA damage in tumor cells; during this treatment, autophagy can act directly or indirectly to regulate genomic stability by modulating the generation of reactive oxygen species (ROS), targeting proteins responsible for double-strand break repair, or mediating the selective degradation of nuclear components. Such multifaceted activity was demonstrated by Chaachouay et al., predominantly in the aggressive PC3 prostate cancer line, where results indicated that the blockade of autophagy (via the silencing of ATG5 and Beclin-1 genes) significantly sensitized the cancer cells to ionizing radiation [109].

Prostate cancer cells have also been investigated regarding autophagy-related processes during chemotherapy. Pickard et al. [110] observed that 3-methyladenine (an autophagy inhibitor that blocks class III PI3K) partially protected PC3 cells from docetaxel-induced cytotoxicity, which was associated with elevated LC3-II levels during the treatment.

Beyond standard chemotherapy, the latest targeted therapies focus on the PI3K/Akt pathway and the PTEN protein, as PTEN mutations are highly prevalent in prostate cancer. Phase III clinical trials evaluating capivasertib (an Akt kinase inhibitor) in combination with abiraterone are currently ongoing for PTEN-deficient metastatic hormone-sensitive prostate cancer [111]. Furthermore, promising preclinical results indicate that the inhibition of Akt by this compound in PC3 cells leads to the modulation of mTOR activities alongside an increase in LC3 flux, which is fundamental to the autophagic process [112].

## 6. Pharmacological Inhibition of Autophagy: From Chloroquine to Novel Agents

The only clinically approved inhibitors of autophagy are chloroquine (CQ) and its derivative, hydroxychloroquine (HCQ). The mechanism of action of these compounds centers on elevating the luminal pH of lysosomes. This alkalization leads to the inactivation of acidic hydrolases, thereby preventing the degradation of engulfed cargo within the lysosome and blocking the final fusion of the autophagosome with the lysosome, effectively suppressing the terminal stage of autophagy. At peak concentrations, hydroxychloroquine exhibits lower toxicity compared to chloroquine, which has prompted its preferential use in clinical trials [113,114]. The therapeutic efficacy of hydroxychloroquine has already been validated in various malignancies, including renal cell carcinoma, estrogen receptor-positive breast cancer, melanoma, and colorectal cancer. In the context of prostate cancer, Phase II clinical trials have confirmed the induction of autophagic cell death following HCQ administration in patients exhibiting hormone-dependent prostate-specific antigen (PSA) progression post-local therapy. McCarthy et al. [115] hypothesized that the inhibition of autophagy by hydroxychloroquine contributes to the suppression of tumor cell growth. In a cohort of patients previously treated for prostate cancer, an analysis of PSA levels before and after the initiation of HCQ therapy confirmed that HCQ possesses clinical activity that impacts prostate cancer progression.

Furthermore, hydroxychloroquine has emerged as a potent stimulator of the tumor suppressor protein PAR-4 (Prostate Apoptosis Response-4), which selectively induces apoptosis in malignant cells while sparing healthy tissues. Phase II clinical trials demonstrated that in approximately 60% of patients, PAR-4 protein levels increased by at least 50%, a change that correlated directly with a reduction in PSA levels. Notably, after approximately 12 months of follow-up, 37% of these patients successfully avoided the need for androgen deprivation therapy (ADT) [116].

In a preclinical study of metastatic prostate cancer utilizing LNCaP and PC-3 cell lines, a palladium(II) barbiturate complex was administered as a cytotoxic agent, with chloroquine applied to block autophagic “rescue” from apoptosis. It was observed that tumor cells, driven toward cell death pathways due to DNA damage inflicted by the palladium complex, had their alternative survival route (autophagy) effectively blocked by the addition of chloroquine. This dual targeting forces the malignant cells into definitive apoptosis, a mechanism corroborated by elevated levels of pro-apoptotic proteins, modulations in the expression of PI3K/Akt/mTOR pathway-related proteins, and the characteristic translocation of phosphatidylserine observed during apoptosis [117].

In a study conducted by Saleem et al. [118], the application of ABT-737 (a BH3 domain mimetic) in combination with hydroxychloroquine revealed that this mimetic, in addition to antagonizing anti-apoptotic proteins, induces massive autophagy, evidenced by the relocalization of the autophagosomal marker LC3 and a reduction of p62 in tumor tissue. When combined with hydroxychloroquine, ABT-737 triggers the generation of substantial quantities of reactive oxygen species (ROS), which synergistically exacerbates the mortality of prostate cancer cells.

Despite their favorable effects against prostate cancer cells, these compounds present certain translational limitations. Primarily, the majority of the studies have been conducted in vitro, and the equivalent dosages required for human efficacy may manifest high toxicity, most notably cardiotoxicity and the development of retinopathy. Furthermore, chloroquine and hydroxychloroquine act systemically, thereby inhibiting autophagy not only in malignant cells but also in healthy tissues, where it is essential for the repair and clearance of damaged organelles; this non-specific blockade can consequently lead to unintended cytotoxicity in healthy cells.

Among the most recently identified and highly promising targets currently being evaluated in prostate cancer cell lines are the VPS34 kinase and the Atg4B protease. Regarding Atg4B, recent findings indicate that the application of specific inhibitors targeting this enzyme effectively suppresses autophagy induced by therapeutic agents against castration-resistant prostate cancer (CRPC) and concurrently enhances apoptosis [119]. Additionally, research by Kudo et al. [120] investigating long-chain fatty acids as potential modulators of Atg4B function demonstrated that these acids, particularly docosahexaenoic acid (DHA), exhibit the most potent inhibitory activity against autophagy induced by androgen receptor signaling inhibitors (ARSI) in LNCaP cells, leading to increased apoptotic cell death. These results suggest that DHA supplementation may improve the therapeutic outcomes of prostate cancer treatments.

VPS34 kinase inhibitors are designed to block the activity of VPS34, thereby preventing the formation of early autophagic vesicles. Studies utilizing DU145 and PC3 cell lines have demonstrated that the pharmacological inhibition of this kinase profoundly modulates the tumor microenvironment by stimulating the secretion of critical chemokines, such as CCL5 and CXCL10. This chemokine profile promotes the infiltration and enhanced activity of T lymphocytes within the tumor bed. Through this immunomodulatory mechanism, the blockade of VPS34 exhibits robust synergy with immunostimulatory therapies (e.g., immune checkpoint inhibitors targeting the PD-1/PD-L1 axis), effectively sensitizing immunologically “cold” tumors to the host’s immune response [121].

The initial stages of autophagy can also be blocked using ULK-1 kinase inhibitors (e.g., SBI-0206965). Preclinical studies have demonstrated that the administration of SBI-0206965 in combination with enzalutamide effectively overcomes castration resistance in prostate cancer (CRPC) cells, ultimately leading to apoptosis [122]. An innovative compound in this context is Spautin-1, which enhances the degradation of the Beclin-1 protein complex—a crucial element in autophagosome formation. Although its ability to sensitize tumors to oxidative stress via ROS-mediated DNA damage was primarily demonstrated in melanoma models [123], this mechanism provides a strong rationale for analogous applications in prostate cancer. Spautin-1 antagonizes the activity of ubiquitin-specific protease 10 and ubiquitin-specific protease 13, which are responsible for removing ubiquitin from the BECN1 complex. Consequently, Spautin-1 increases the proteasome-mediated degradation of the BECN1 complex, which ultimately reduces autophagosome formation and inhibits relatively early events in the autophagic pathway.

Alternatives to HCQ that yield superior efficacy within the tumor microenvironment include Lys05 and ROC-325. Lys05 is a water-soluble agent that profoundly accumulates in the lysosomes of prostate cancer cells, thereby exhibiting a more potent antitumor effect in in vivo models. In the context of CRPC, the application of Lys05 allows for the circumvention of castration resistance, particularly when combined with agents such as enzalutamide. The mechanism underlying this combination therapy relies on blocking the androgen receptor with a specific drug (e.g., enzalutamide) while concurrently administering Lys05. By accumulating in the lysosomes, Lys05 inhibits autophagy and forces the cell into apoptosis [124]. ROC-325, on the other hand, is an oral lysosomal inhibitor currently investigated in combination with chemotherapeutics. Although its profound capacity to disrupt autophagic degradation and antagonize tumor pathogenesis has been initially validated in renal cell carcinoma [125], its mechanistic potential is highly applicable to other solid tumors. In resilient cancer cells, such lysosomal inhibition profoundly impairs protein repair capacity. When translated to prostate cancer models, combining such agents with standard therapies significantly hinders tumor cell DNA repair and enhances radiosensitivity [126].

Furthermore, bortezomib acts as a proteasome inhibitor. The blockade of this complex forces the cell to rely exclusively on the autophagic process for survival. Consequently, the subsequent administration of autophagy inhibitors decisively leads to cancer cell death. In the context of the androgen receptor (AR) involved in prostate cancer development, bortezomib inhibits the proteasome, leading to the disruption of intracellular signaling. This impedes the proper nuclear translocation of the AR, thereby attenuating tumor proliferation [127].

## 7. Preclinical Models in Prostate Cancer Research

Implementing effective therapies in patients remains a formidable challenge, even for modern medicine. Cellular models, despite their substantial contribution to the development of novel pharmaceuticals, possess significant limitations that impede the market introduction of new drugs. These limitations encompass the inability to perfectly replicate the three-dimensional architecture of tissues and organs, the native tumor microenvironment, and the blood-brain barrier. Currently, several notable in vivo models are utilized to bridge this gap, including CDX, PDX, GEMMs, and humanized mouse models.

The cell line-derived xenograft (CDX) model is generated by implanting human cancer cell lines into immunodeficient mice to establish tumors in vivo. The advantages of this model include the simplicity of cancer cell implantation and the rapid generation of a large number of tumor-bearing animals. Furthermore, the integration of in vitro cell culture stages enables high-throughput model production and shortens tumor latency, thereby enhancing the efficiency of oncological drug development. Conversely, a major disadvantage is the potential loss of tumor heterogeneity and the subsequent lack of a stable tumor microenvironment.

The patient-derived xenograft (PDX) model, established directly from tumor tissues excised from a patient, overcomes some of these issues. The primary advantages of this model include a superior recapitulation of tumor heterogeneity, its native microenvironment, and its three-dimensional architecture. The generation of these models inherently requires immunodeficient mice [128,129].

Genetically engineered mouse models (GEMMs), on the other hand, are transgenic mice harboring specific mutations designed to mimic the oncogenic activation and/or tumor suppressor loss observed in specific human malignancies. The tumors developing in these models accurately reflect human cancers at the histopathological, molecular, and clinical levels [130]. However, the drawbacks of these models include prolonged experimental timelines and intrinsic differences between murine and human biology, meaning the tumor arises in a somewhat ‘artificial’ context. Humanized mouse models are generated through various processes, such as the introduction of foreign DNA molecules and their random integration into the murine genome, or through the targeted inactivation or insertion of specific genes (knock-out and knock-in mice).

Tumors generated in GEMMs are primarily utilized to analyze the functions of genes involved in tumorigenesis and precancerous states, to characterize the tumor microenvironment, and to test novel oncological therapies. In terms of overall utility, precise analysis of tumorigenesis starting from the molecular level, and accurate recapitulation of the tumor and its microenvironment, the humanized mouse model appears to be the most superior. Consequently, it is extensively employed in the design of cutting-edge immunotherapies and cell-based therapies (e.g., CAR-T). Ultimately, while CDX and PDX models are often utilized for fundamental toxicity assessments and the elucidation of cellular drug mechanisms, GEMMs remain particularly valuable for evaluating immunotherapies and the early stages of carcinogenesis [130,131,132].

## 8. Biological and Clinical Significance

An analysis of the currently available scientific literature clearly demonstrates that the role of autophagy in prostate cancer is highly complex and frequently characterized as a “double-edged sword.” On the one hand, this process exerts cytoprotective functions, enabling malignant cells to survive under adverse conditions, such as oxidative stress or androgen deprivation. On the other hand, appropriately targeted autophagic flux can trigger cell death (cytotoxic autophagy), thereby presenting novel therapeutic opportunities. A principal conclusion drawn from this literature review is that standard therapeutic modalities—such as androgen deprivation therapy (ADT), chemotherapy (e.g., docetaxel), and radiotherapy—frequently induce autophagy themselves. In prostate cancer cell lines (e.g., LNCaP or PC3), autophagy emerges as a critical escape mechanism utilized by the tumor to evade therapeutic-induced death. Consequently, the implementation of combination therapies—specifically, integrating a standard therapeutic agent with an autophagy inhibitor—represents a highly logical and promising approach. Preclinical and clinical studies utilizing chloroquine (CQ) and hydroxychloroquine (HCQ) have yielded encouraging results, demonstrating significant tumor sensitization to treatment. However, the primary translational barrier in applying these findings to patients lies in the associated adverse effects. HCQ operates non-selectively, effectively inhibiting autophagy in healthy tissues as well, which can lead to severe toxicities, including cardiotoxicity and retinopathy. Therefore, the future of PCa management likely resides not in broad-spectrum agents like HCQ, but in more precise molecular targets, such as the VPS34 kinase or the Atg4B enzyme.

Vps34, acting as a lipid kinase, converts specific lipids within cell membranes to generate PI(3)P, which serves as a recruitment platform for other proteins, thereby facilitating autophagosome formation [133]. VPS34 inhibitors have been significantly optimized over the years—ranging from first-generation agents (represented by 3-methyladenine and wortmannin), which are highly toxic and unsuitable for clinical use, to second-generation compounds, including SAR405, VPS34-IN1, and SB02024. SAR405 is a highly targeted inhibitor; when co-administered with everolimus, it induces synthetic lethality in tumors, thereby preventing cancer cells from utilizing autophagy as a survival mechanism. VPS34-IN1 is another highly precise Vps34 inhibitor that has been tested across various solid tumor models, yielding highly promising results [134]. SB02024 represents the latest class of compounds in this group; it demonstrates potent in vivo antitumor activity by sensitizing resistant cancer cells to chemotherapy [121].

ATG4B is a cysteine protease involved in LC3 protein processing. The discovery of ATG4B inhibitors was preceded by extensive experimental efforts, as this protease performs dual, opposing roles in the autophagic process. Key ATG4B inhibitors include tioconazole, NSC185058, UAMC-2526, S130, and FMK-9. Tioconazole is a well-known pharmacological agent traditionally used as an antifungal; in the context of cancer cell research, it was discovered to bind directly to the active site of ATG4B, completely blocking its activity [135]. NSC185058 suppresses autophagy without affecting the mTOR kinase. Currently, UAMC-2526 is the most potent inhibitor in this class; it enhances the efficacy of oxaliplatin in treatment-resistant tumors [136]. S130 and FMK-9 operate by enforcing apoptosis through the accumulation of damaged proteins [137].

Furthermore, palmitoyl-protein thioesterase 1 (PPT1) inhibitors, such as DC661 and DQ661, warrant mention as they specifically target the PPT1 enzyme. Its deactivation halts autophagy and concurrently suppresses mTOR kinase activation, thereby depriving cancer cells of growth-stimulating signals [138].

Inhibitors of p97 also constitute a significant group of compounds, as the proteins they inhibit are responsible for the transport of damaged proteins and the maturation of autophagosomes. A prime example is CB-5083, which has been evaluated in Phase I clinical trials for solid tumors, confirming the hypothesis that directly targeting the transport pathways of damaged cellular components is highly toxic to cancer cells [139].

Current oncological research and treatment strategies are shifting towards Vps34 and ATG4B inhibitors, moving away from chloroquine-based drugs and their derivatives due to a critical factor: the generation of substantial cellular stress. Chloroquine-based therapies promote autophagosome formation but block their final degradation, leading to a massive accumulation of autophagosomal vesicles and inducing unnecessary cellular stress that also affects healthy cells. Conversely, with Vps34 or ATG4B inhibitors, the cell is cut off from building blocks and deprived of the ability to initiate autophagy even before autophagosome formation begins.

Synthesizing these findings from the literature review, a certain contradiction in the research regarding autophagy in prostate cancer becomes apparent. The majority of in vitro experiments indicate that autophagy inhibition sensitizes CRPC cells to treatment (e.g., with enzalutamide); however, in vivo studies and early-stage clinical trials often yield conflicting results. This discrepancy in the literature stems from the fact that early studies often overlooked the metabolic differences of cancer cells and failed to account for the tumor microenvironment (TME). For instance, the blockade of a single pathway may trigger compensatory survival signaling or induce systemic toxicity across the tumor cell network.

Another crucial aspect addressed in this paper is the regulatory role of miRNA molecules, which represent highly attractive targets for personalized medicine. The ability to detect specific circulating miRNAs—such as miR-21, miR-141, and miR-375—in patient serum offers profound potential for the development of liquid biopsies. This approach could facilitate the much earlier detection of metastasis or therapeutic resistance, circumventing the need for invasive tissue biopsies. Nevertheless, it must be acknowledged that the majority of miRNA research remains in its nascent stages, relying predominantly on in vitro cell line models. In vivo, the network of interactions between miRNAs and their target mRNAs (e.g., for Bcl-2 family proteins or the mTOR pathway) is vastly more intricate. Furthermore, identical miRNA molecules may exert divergent functions depending on the stage of the disease, presenting a significant challenge in designing a single, universal therapeutic based on antagomiRs or miRNA mimics.

Finally, recent reports exploring the integration of autophagy inhibitors with immunotherapy present a highly intriguing frontier. Prostate cancer is traditionally classified as an “immunologically cold” tumor, indicating a poor clinical response to immune-stimulating agents (e.g., anti-PD-L1 inhibitors). The inhibition of autophagy, by inducing metabolic stress within the tumor microenvironment and stimulating T-cell infiltration, possesses the potential to reverse this state. However, this domain requires extensive further preclinical investigation to ensure that the blockade of autophagy within the tumor does not simultaneously impair the functional integrity of the infiltrating T lymphocytes, which also inherently rely on autophagic processes for their survival and effector functions.

Immune evasion remains a formidable hurdle in eradicating malignant cells. Yamamoto et al. demonstrated in pancreatic cancer models that highly aggressive tumor phenotypes selectively upregulate autophagy—specifically via the NBR1 cargo receptor—to selectively capture and degrade major histocompatibility complex class I (MHC-I) molecules. This lysosomal degradation of surface antigens allows the tumor to effectively evade cytotoxic T-lymphocyte surveillance, representing a universal immune-escape mechanism highly relevant to solid tumors, including prostate cancer [140]. Conversely, in gastric cancer studies by Wang et al., the pharmacological blockade of autophagy triggered the accumulation of the multifunctional adaptor protein p62, which paradoxically upregulated the immune-checkpoint ligand PD-L1 due to persistent cellular stress. Consequently, the concurrent administration of an autophagy inhibitor with anti-PD-L1 antibodies synergistically redirected the cells toward definitive apoptosis, highlighting a therapeutic strategy with profound translational potential for advanced prostate cancer [141].

These findings reveal a distinct contradiction in the current literature regarding the interplay between autophagy and the immune system. While inhibiting autophagy in cancer cells prevents MHC-I degradation and enhances the efficacy of immune checkpoint inhibitors, the systemic administration of autophagy inhibitors impairs T-cell function and promotes myeloid-derived suppressor cells (MDSCs), which could ultimately protect the tumor from destruction. This dual action creates a scenario where unequivocally analyzing the literature data becomes highly challenging.

The role of autophagy is undoubtedly bidirectional; the specific characteristics of the tumor, its microenvironment, and the therapeutic targeting are of paramount importance here. Autophagy can augment the antitumor immune response by supporting specific T-cell subsets and promoting tumor antigen presentation. Simultaneously, however, it can facilitate cancer cell survival by degrading tumor antigens, enhancing regulatory T cells (Tregs), and supporting MDSCs.

In the context of prostate cancer, autophagy modulation primarily involves Sigma-1 inhibitors (small-molecule Sigma-1 modulators can be utilized to regulate PD-L1 in cancer cells and stimulate its degradation via selective autophagy), short-chain fatty acids (SCFAs derived from the gut microbiota in CRPC promoted cancer progression by inducing cancer cell autophagy), or an oncolytic adenovirus combined with temozolomide (the combination of an oncolytic adenovirus with low-dose temozolomide (TMZ) and metronomic cyclophosphamide increased cancer cell autophagy and elicited an antitumor immune response) [142].

### Future Perspectives: Overcoming Translational Barriers in Autophagy Inhibition

The primary challenge currently facing experimental oncology is the safe combination of autophagy inhibitors with immunotherapy. Because this process is crucial for both tumor survival and proper T-lymphocyte function, the first and most critical experimental question becomes achieving so-called spatial separation. Methodologically, research in this field should shift towards targeted drug delivery systems (DDS). Instead of the systemic administration of non-selective blockers, the focus should be placed on carriers (e.g., liposomes) that release autophagy inhibitors exclusively within the specific, acidic, or hypoxic tumor microenvironment (TME). The efficacy and safety of such solutions are best validated using advanced 3D co-culture models, directly assessing the viability of cancer cells relative to peripheral blood mononuclear cells (PBMCs).

If spatial targeting encounters technological barriers, another pathway in the research roadmap must be precise treatment scheduling, namely temporal separation. Preclinical animal models with an intact immune system (e.g., humanized mice) are essential to determine the optimal sequence of drug administration. Rather than concurrent therapy, a highly promising strategy appears to be tumor priming—a short-term, pulse administration of an autophagy inhibitor to prevent the degradation and promote the accumulation of MHC-I molecules on the tumor surface. Subsequently, following an appropriately optimized washout phase, anti-PD-L1 antibodies can be safely introduced, thereby circumventing the risk of damaging activated lymphocytes.

The third pillar of this preclinical strategy is the identification of predictive biomarkers of response. Prior to entering clinical trials, it is imperative to utilize techniques such as spatial transcriptomics to compare tissue profiles between responding and non-responding animal cohorts. Researchers should particularly focus on the correlation between basal lymphocytic infiltration and the degree of intracellular MHC-I restoration. From a translational perspective, investigations should also encompass specific miRNA signatures and markers of autophagic flux disruption (e.g., p62 protein levels) that can be detected via liquid biopsy. In the future, this will facilitate the rapid and non-invasive stratification of patients into appropriate therapeutic cohorts.

## 9. Conclusions

Future research in castration-resistant prostate cancer (CRPC) must leverage advanced 3D heterotypic co-cultures to accurately model the TME, alongside targeted nanocarrier delivery systems to restrict the action of autophagy modulators directly to the tumor site. This targeted approach is vital for minimizing off-target effects and preserving T-cell viability. Furthermore, utilizing analytically validated liquid-biopsy biomarkers will be essential for dynamic patient stratification and disease monitoring.

Given that cytoprotective autophagy underpins adaptive resistance to ADT, chemotherapy and radiation, future therapeutic strategies must combine standard-of-care treatments, such as abiraterone acetate, with novel proximal autophagy inhibitors. Mechanistically, rationally targeting this autophagic flux presents a strong theoretical and preclinical rationale to help dismantle TME immunosuppression and sensitize “cold” tumors to immune checkpoint blockade (ICB), though these findings still require robust clinical validation.

## Data Availability

No new data were created or analyzed in this study. Data sharing is not applicable to this article.

## Abbreviations

The following abbreviations are used in this manuscript:

ADT	Androgen Deprivation Therapy
Akt/PKB	Protein Kinase B
AMPK	AMP-activated protein kinase
AR	Androgen Receptor
ARSI	Androgen Receptor Signaling Inhibitors
BPH	Benign Prostatic Hyperplasia
CAR-T	Chimeric Antigen Receptor T-cell
CDX	Cell line-Derived Xenograft
CMA	Chaperone-Mediated Autophagy
CQ	Chloroquine
CRPC	Castration-Resistant Prostate Cancer
DAPK	Death-Associated Protein Kinase
DDR	DNA Damage Repair
DDS	Drug Delivery Systems
DHA	Docosahexaenoic Acid
DISC	Death-Inducing Signaling Complex
EMT	Epithelial-Mesenchymal Transition
GEMM	Genetically Engineered Mouse Models
HCQ	Hydroxychloroquine
HIF-1α	Hypoxia-Inducible Factor 1-alpha
JNK	c-Jun N-terminal kinase
LAMP-2A	Lysosome-Associated Membrane Protein type 2A
lncRNA	Long non-coding RNA
MAPK	Mitogen-Activated Protein Kinase
mCRPC	Metastatic Castration-Resistant Prostate Cancer
MDSC	Myeloid-Derived Suppressor Cells
MHC-I	Major Histocompatibility Complex class I
miRNA	MicroRNA
mTOR	Mammalian Target of Rapamycin
ncRNA	Non-coding RNA
NEPC	Neuroendocrine Prostate Cancer
PBMC	Peripheral Blood Mononuclear Cells
PCa	Prostate Cancer
PDX	Patient-Derived Xenograft
PI3K	Phosphoinositide 3-kinase
pre-miRNA	Precursor miRNA
pri-miRNA	Primary miRNA
PSA	Prostate-Specific Antigen
PTEN	Phosphatase and Tensin Homolog
RISC	RNA-induced Silencing Complex
ROS	Reactive Oxygen Species
SCFA	Short-Chain Fatty Acids
TME	Tumor Microenvironment
TMZ	Temozolomide
TRAIL	Tumor Necrosis Factor-Related Apoptosis-Inducing Ligand
Treg	Regulatory T cells
